# The association between 25-hydroxyvitamin D and parathyroid hormone in adolescents living with HIV in southern Africa: a cross-sectional study

**DOI:** 10.1017/S0007114525000509

**Published:** 2025-04-14

**Authors:** Tafadzwa Madanhire, Kate A. Ward, Amy MacDougall, Nuredin I. Mohammed, Lackson Kasonka, Hildah B. Mabuda, Molly Chisenga, Jonathan C. Y. Tang, William D. Fraser, Tsitsi Bandason, Nyasha V. Dzavakwa, Victoria Simms, Rashida A. Ferrand, Celia L. Gregson

**Affiliations:** 1 The Health Research Unit Zimbabwe, Biomedical Research and Training Institute, Harare, Zimbabwe; 2 MRC Lifecourse Epidemiology Centre, Human Development and Health, University of Southampton, Southampton, UK; 3 Department of Infectious Disease Epidemiology, Faculty of Epidemiology and Population Health, London School of Hygiene and Tropical Medicine, London, UK; 4 MRC International Statistics and Epidemiology Group, Faculty of Epidemiology and Population Health, London School of Hygiene & Tropical Medicine, London, UK; 5 Medical Research Council Unit The Gambia at the London School of Hygiene & Tropical Medicine, Banjul, The Gambia; 6 University Teaching Hospital, Lusaka, Zambia; 7 Bioanalytical Facility, Norwich Medical School, University of East Anglia, Norwich Research Park, Norwich, UK; 8 Clinical Biochemistry, Diabetes and Endocrinology, Norfolk and Norwich University Hospital, Norwich, UK; 9 Clinical Research Department, Faculty of Infectious and Tropical Diseases, London School of Hygiene and Tropical Medicine, London, UK; 10 Global Musculoskeletal Research Group, Musculoskeletal Research Unit, Bristol Medical School, University of Bristol, Bristol, UK

**Keywords:** Vitamin D, Parathyroid hormone, Adolescent, Africa, HIV

## Abstract

Low vitamin D associated with high parathyroid hormone (PTH) is common in HIV infection. We determined the association between total 25(OH)D and PTH in adolescents living with HIV, in Zambia and Zimbabwe. Adolescents (11–19 years) perinatally infected with HIV and established on antiretroviral therapy for ≥ 6 months were recruited into a cross-sectional study. Socio-demographic and clinical characteristics were recorded, anthropometry measured and fasted serum concentrations of 1,25(OH)_2_D, total 25(OH)D and intact PTH measured. The association between total 25(OH)D and PTH was examined using natural cubic spline regression. 842 participants (female: 53·2%) with a median age of 15·5 (IQR: 13·2–17·9) years were enrolled. Median antiretroviral therapy duration was 9·8 (IQR: 6·3–12·3) years, and 165/841 had an HIV viral-load >60 copies/ml. Stunting (height-for-age z-score <–2) and underweight (weight-for-age z-score <–2) were observed in 29·9 and 30·0%, respectively. Three-quarters reported daily Ca intakes <150 mg/d. The mean (sd) concentrations of total 25(OH)D and 1,25(OH)_2_D were 66·1(16·5) nmol/l and 210·6 (70·4) pmol/l, respectively, and median PTH level was 4·3 (IQR: 3·3–5·5) pmol/l. There was an inverse non-linear relationship between total 25(OH)D and PTH, 25(OH)D levelling off at 74·6 nmol/l (95 % CI: 74·5, 75·2). Results were consistent in those taking tenofovir disoproxil fumarate and virally unsuppressed participants. In this population with extremely low habitual Ca intakes, the lack of association between 25(OH)D and PTH when 25(OH)D exceeded 75 nmol/l potentially suggests that levels of 25(OH)D >75 nmol/l may need to be achieved to improve bone health; investigation is needed in future research studies.

What constitutes ‘adequate’ vitamin D is debated around the world^(1–3)^. There is no global consensus on the definition of 25-hydroxyvitamin D (25(OH)D) deficiency, with suggested values ranging between 30 and 100 nmol/l^(4–6)^. 25(OH)D regulates skeletal mineralisation during growth and is also thought to play an important role in facilitating macrophage and T-cell function and maintaining a healthy gut microbiome^(7–9)^. In children and adolescents, vitamin D deficiency is associated with secondary hyperparathyroidism, rickets, osteomalacia and poor bone growth^(10)^.

Fifty-three percent of individuals with HIV live in Eastern and Southern Africa^(11)^, a region characterised by a predominantly temperate climate, providing ample sunshine throughout the year^(12,13)^. Low vitamin D is commonly reported among people living with HIV^(14,15)^ in part, it is thought, due to certain antiretroviral drugs^(16)^. Tenofovir disoproxil fumarate is associated with lower 25(OH)D, thought to result from the upregulation of 24-hydroxylase, leading to lower circulating 25(OH)D and 1,25-dihydroxyvitamin D (1,25(OH)_2_D) concentrations^(16,17)^. To date, studies on the prevalence of vitamin D deficiency in children and adolescents with HIV have generated variable estimates, mainly due to varying thresholds for insufficiency^(18–20)^.

In 2018, a global systematic review of vitamin D deficiency in children and adolescents living with HIV concluded that the literature comprised multiple small, underpowered and heterogenous vitamin D studies from which it was not possible to draw a firm conclusion on what constitutes an adequate concentration of 25(OH)D for optimising bone health, lowering the risk of secondary hyperparathyroidism and prevention of rickets and osteomalacia^(21)^.

Methods of modelling the relationship between 25(OH)D and parathyroid hormone (PTH) have so far been limited to linear spline models^(22–24)^ and non-linear locally weighted regression smoothing (loess)^(25–27)^. However, the use of linear spline models is questionable since the relationship between 25(OH)D and PTH is non-linear, regardless of the method of segmentation. Similarly, besides its flexibility and ability to show a pattern of association between two variables, a loess function requires dense data to give a smoothed estimate such that they lack tail precision when data are sparse^(28,29)^.

This study aimed to determine the concentration of 25(OH)D at which the association with PTH changes, in children and adolescents living with HIV in Zimbabwe and Zambia. Understanding such relationships may provide insights into what might constitute ‘adequate’ vitamin D in the context of HIV infection, chronically low habitual Ca intakes and in turn an understanding of musculoskeletal development in peripubertal adolescents growing up with HIV.

## Methods

### Study design, setting and population

We conducted a cross-sectional study nested within a phase III individually randomised, double-blinded, placebo-controlled trial of vitamin D_3_/Ca carbonate or placebo (vitamin D for adolescents with HIV to reduce musculoskeletal morbidity and immunopathology (VITALITY): Pan African Clinical Trials Registry PACTR20200989766029)^(30)^.

The trial enrolled 842 
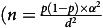
 , where *p* is the prevalence of vitamin D deficiency (< 30 nmol/l)^(31)^, *α* is the 95 % confidence level (1·96) and *d* is the error (3 %)) adolescents aged 11–19 years living with HIV recruited from public sector HIV outpatient clinics in Harare, Zimbabwe and Lusaka, Zambia, between January and December 2021. Lusaka and Harare have relatively similar latitudes of –15·4° and –17·8°, respectively, suggesting comparable sunlight exposure^(32)^. The inclusion criteria were perinatally acquired HIV, taking antiretroviral therapy (ART) for at least 6 months and being willing to give blood samples. Exclusion criteria included being acutely unwell, taking tuberculosis treatment, currently pregnant or breastfeeding and a history of either thyrotoxicosis, chronic renal disease, hypercalcemia, phosphate metabolism disorder or osteomalacia. Baseline data were used for this analysis.

### Data collection

An interview-administered questionnaire pre-programmed using an Open Data Kit on electronic tablets was used to collect socio-demographic and clinical data including HIV history. Socio-economic status quintiles were derived from a principal component analysis of the participant’s household assets. Dietary Ca intake was assessed using a dietary diversity questionnaire adopted on that of the FAO of the UN questionnaire adapted to Zimbabwe and Zambia and focussing on multi-micronutrient-rich foods (e.g. dairy products, eggs, fish, legumes)^(33)^. Using the FAO food composition tables, estimated dietary Ca intake was then calculated based on the International Osteoporosis Foundation frequency of consumption, serving size and Ca quantity per portion size (online Supplementary Table 1)^(34–36)^.

Participants underwent height and weight measurements and Tanner pubertal staging measurement; Z-scores were calculated using UK reference data^(37–39)^. Stunting and underweight were classified as height- and weight-for-age Z-scores < –2, respectively^(38)^. Venous blood was collected into EDTA tubes (BD Vacutainer) for 1,25(OH)_2_D, total 25(OH)D and intact PTH measurements. Blood tubes were promptly centrifuged, and aliquots were stored at –20°C, with all analyses performed on the first thaw. HIV viral load testing was performed using the Qiagen rotor gene Q, Hologic Panther or GeneXpert machines in Zambia and the Roche COBAS AmpliPrep/COBAS TaqMan48 in Zimbabwe. The classification of HIV viral load suppression (< 60 *v*. ≥ 60 copies/ml) was based on the assay limit of detection.

### Total 25(OH)D, 1,25(OH)_2_D, 24,25(OH)_2_D and intact PTH measurements

25(OH)D, 1,25(OH)_2_D and intact PTH concentrations were analysed at the Bioanalytical Facility, University of East Anglia (Norwich, UK). Liquid chromatography-tandem MS methods were used for 25(OH)D and 1,25(OH)_2_D as previously described^(40,41)^. The 25(OH)D3 and 25(OH)D2 assays were calibrated using the National Institute of Science and Technology standard reference material SRM972a. Inter-assay CV was < 8·4 % across the assay working range of 0·1 to 200·0 nmol/l. 1,25(OH)_2_D3, 1,25(OH)_2_D2, 24,25(OH)_2_D3 and 24,25(OH)_2_D2 were analysed by liquid chromatography-tandem MS following immunoaffinity sample pre-treatment and derivatisation. The assays were calibrated using certified pure internal standards (Cerilliant, LGC). Inter-assay CV was < 9·8 % across the assay working range of 20·0–800·0 pmol/l. All vitamin D metabolite assays met the requirements specified by the vitamin D external quality assessment (DEQAS) scheme (http://www.deqas.org/; accessed on 30 Oct 2023). The 25(OH)D3 and 25(OH)D2 assays showed < 6 % accuracy bias against the Centers for Disease Control and Prevention reference measurement procedure target values on the DEQAS scheme. Intact PTH was analysed by electrochemiluminescence immunoassay on the COBAS (Roche Diagnostics) platform. The inter-assay CV was ≤ 3·8 % across the analytical range of 0·1–530·0 pmol.

### Statistical analysis

Data were cleaned, checked and analysed using RStudio (2023: v.421; Integrated Development for R.)^(42)^. All quantitative variables were summarised using mean ± sd if normally distributed, or otherwise as median with an interquartile range (IQR). Categorical variables were summarised as frequencies with percentages. Participant age, dietary Ca intake, PTH and 1,25(OH)_2_D distributions were summarised by Tanner stage and sex. Monthly variation in 25(OH)D concentrations over the data collection period was investigated using box (median and IQR) plots. Correlations between 1,25(OH)_2_D and PTH, 25(OH)D and dietary Ca intakes were determined using scatter plots.

The analysis was conducted sequentially: (i) explored the relationship between total 25(OH)D and PTH using a scatter plot with a non-parametric, loess line fitted. As the association exhibited different slopes in different ranges of the data, piecewise regression was used to model the relationship between total 25(OH)D and PTH; (ii) considered different univariable piecewise regression models ranging from linear to order-six polynomial functions for total 25(OH)D and PTH to best fit the data and determine the slope pattern; (iii) used the likelihood ratio test to identify the best fitting univariable piecewise regression model; (iv) identified a natural cubic spline regression as the best model fitting the relationship between total 25(OH)D and natural log-transformed PTH (see online Supplementary Methods)^(43)^; (v) further used the Akaike information criterion to determine the optimal df for the natural cubic spline regression curve (range of df (2–6)); (vi) assessed the slope pattern by plotting the piecewise natural cubic spline regression coefficient against total 25(OH)D to determine an inflection point (a point where the relationship between 25(OH)D and PTH differed before and after that point)^(44)^; (vii) identified an inflection point in the cubic spline regression curve where the association between 25(OH)D and PTH levelled off, as informed by the 95 % CI of the regression coefficient.

In sensitivity analyses, the study first assessed the natural cubic spline model stratified by (i) ART regimen (those taking tenofovir disoproxil fumarate *v*. those not) and (ii) HIV viral load (those < and > 60 copies/ml) to determine the consistency of inflection points. Second, a natural cubic spline model was fitted for the relationship between the vitamin D metabolic ratio (25(OH)D/24,25(OH)_2_D) and 25(OH)D to confirm the consistency of the inflection point where the association between 25(OH)D and the vitamin D metabolic ratio levelled off^(27)^.

## Results

### Participant characteristics

We enrolled 842 participants, with median age of 15·5 (IQR: 13·2–17·9) years, and 53·2 % female (*n* 448) (Table 1). Most participants were in Tanner stages IV (*n* 207; 24·6 %) and V (*n* 261; 31·1 %) with a higher proportion of girls in the latter category (Tanner stage V: 38 % *v*. 23·2 %) (online Supplementary Table 2). Stunting was common, occurring in 29·9 % of participants (*n* 251/840), as was being underweight (*n* 253/842; 30 %). Three-quarters (*n* 639; 75·9 %) reported consuming no more than 150 mg of dietary Ca per d. The median duration of ART was 9·8 (IQR: 6·3–12·3) years: 81·7 % (*n* 688) were taking a tenofovir disoproxil fumarate containing ART regimen. Overall, 164 of 841 (19·5 %) were virally unsuppressed with an HIV viral load ≥ 60 copies/ml.

**Table 1.** Baseline descriptive characteristics (Numbers and percentages; mean values and standard deviations; median values and interquartile ranges)

	Total subjects (*n* 842)		Zambia (*n* 420)		Zimbabwe (*n* 422)	
	*n*	%	*n*	%	*n*	%
Socio-demographic characteristics						
Sex (female), *n* (%)	448	53·2	230	54·8	218	51·7
Age						
Median	15·5		15·0		16·2	
IQR	13·2–17·9		13·0–17·4		13·6–18·1	
Socio-economic status quintiles, *n* (%)						
Q_1_	170	20·2	80	19·0	90	21·3
Q_2_	167	19·8	86	20·5	81	19·2
Q_3_	175	20·8	101	24·0	74	17·5
Q_4_	162	19·2	77	18·3	85	20·1
Q_5_	168	19·9	76	18·1	92	21·8
Daily dietary Ca intake/d (mg), *n* (%)						
< 150	639	75·9	321	76·4	318	75·4
150–299	129	15·3	66	15·7	63	14·9
300+	74	8·8	33	7·9	41	9·7
Growth characteristics						
Tanner stage, *n* (%)						
I	77	9·2	31	7·4	46	11·0
II	129	15·4	72	17·1	57	13·6
III	166	19·8	90	21·4	76	18·1
IV	207	24·6	102	24·3	105	25·0
V	261	31·1	125	29·8	136	32·4
Height-for-age z-score < –2, *n* (%)	251/840	29·9	126/420	30·0	126/420	30·0
Weight-for-age z-score < –2, *n* (%)	253	30·0	135	32·1	118	28·0
BMI-for-age z-score < –2, *n* (%)	104/838	12·4	60/420	14·3	44/418	10·5
HIV characteristics						
Viral load (*≥* 60 copies/ml), *n* (%)	164/841	19·5	64/419	15·3	100/422	23·7
ART duration						
Median	9·8		8·3		10·3	
IQR	6·3–12·3		4·6–12·2		7·7–12·3	
TDF containing ART regimen, *n* (%)	688	81·7	360	85·7	328	77·7
Efavirenz containing ART regimen, *n* (%)	54	6·4	18	4·3	36	8·5
Vitamin D metabolites						
	Mean	sd	Mean	sd	Mean	sd
Total 25(OH)D (nmol/l), mean (sd)	66·1	16·5	61·3	14·2	70·8	17·3
1,25(OH)_2_D (pmol/l), mean (sd)	210·6	70·4	208·1	65·5	213·1	75·0
24,25(OH)_2_D (nmol/l), mean (sd)	4·1	1·6	3·7	1·3	4·5	1·8
PTH (pmol/l)						
Median	4·3		4·2		4·4	
IQR	3·3–5·6		3·3–5·4		3·3–5·8	

TDF, tenofovir disoproxil fumarate; ART, antiretroviral therapy; PTH, intact parathyroid hormone.

### Serum 1,25(OH)_2_D 24,25(OH)_2_D total 25(OH)D and PTH

The mean 25(OH)D was 66·1 (sd: 16·5) nmol/l; 25(OH)D was comparable between Zimbabwean (mean: 61·3 (sd: 14·2) nmol/l) and Zambian (mean: 70·8 (sd: 17·3) nmol/l) participants. The mean 1,25(OH)_2_D was 210·6 (sd: 70·4) pmol/l and likewise the distribution was similar in the two countries (Zimbabwe: 213·1 (sd: 74·9) pmol/l *v*. Zambia: 208·1 (65·5) pmol/l). In contrast, serum 24,25(OH)_2_D was higher in Zimbabwe (4·5 (sd: 1·8) nmol/l) than in Zambia (3·7 (sd: 1·3) nmol/l) with an overall mean of 4·1 (sd: 1·6) nmol/l. The distribution of PTH was right skewed, with median 4·3 (IQR: 3·3–5·6) pmol/l, with no differences by country (Table 1 and online Supplementary Fig. 1a). No evidence of seasonal variation in 25(OH)D concentrations was seen as medians (IQR) were similar (online Supplementary Fig. 2a). The study determined moderate positive (*r* = 0·274), very weak positive (*r* = 0·013) and very weak negative (*r* = –0·065) correlations between 1,25(OH)_2_D and PTH, 25(OH)D and dietary Ca intake, respectively (online Supplementary Fig. 3a). Dietary Ca intakes and PTH concentrations were similar by Tanner stage in males and females. Marginally higher 1,25(OH)_2_D concentrations were seen in participants in Tanner stage III (online Supplementary Table 2).

### The association between total 25(OH)D and PTH

**Figure 1.**
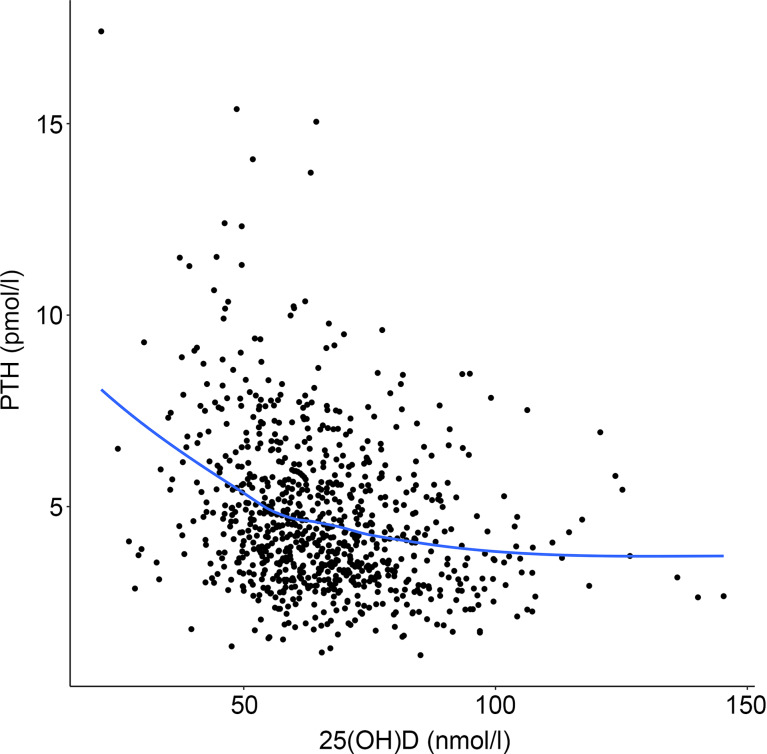
(a) Scatter plot and a non-parametric locally weighted smoothing (loess) fitted line, illustrating the relationship between total 25(OH)D and PTH.

Figure 1 illustrates the relationship between total 25(OH)D and PTH in all participants. The scatterplot (with loess smoother) indicated an inverse non-linear relationship. Notably, the loess line also indicated no discernible change in the concentration of PTH for higher values of 25(OH)D.

### PTH-associated total 25(OH)D inflection points

**Figure 2.**
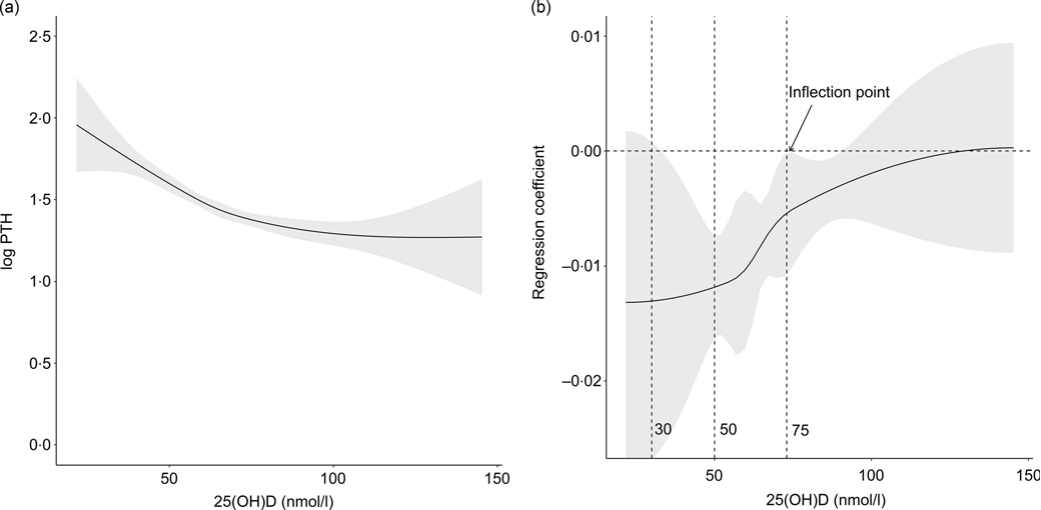
(a) The relationship between total 25(OH)D and PTH (log-transformed), modelled using a non-parametric natural cubic spline curve. The regression curve is fitted with a 95 % CI showing the variation of the natural cubic spline coefficient. (b) Identification of inflection points for the relationship between total 25(OH)D and (log-transformed) PTH at 59·6 and 74·6 nmol/l. The natural cubic spline regression coefficient with a 95 % CI is plotted against total 25(OH)D. Since natural cubic splines involve appropriate partitioning of the curve such that the regression coefficient changes at different values of 25(OH)D, this helps in the identification of inflection points. Commonly used definitions of 25(OH)D deficiency (< 30 nmol/l) and insufficiency (< 50 nmol/l) are shown for illustrative purposes.

The relationship between total 25(OH)D and log-transformed PTH, modelled using a natural cubic spline (Fig. 2(a)), showed a visually similar association pattern to the scatter plot in Fig. 1. Figure 2(b) shows the regression coefficient for the natural cubic spline model (total 25(OH)D *v*. PTH) at different values of 25(OH)D. The model showed a rapid change in the regression coefficient for the natural cubic spline model for values of 25(OH)D from 59·6 nmol/l (95 % CI: 59·4, 59·6) to 74·6 nmol/l (95 % CI: 74·5, 75·2). Figure 2(b) also shows that the association of total 25(OH)D and log-transformed PTH levels off (inflection point) at 74·6 nmol/l (95 % CI: 74·5, 75·2), which is also the point at which the 95 % CI of the regression coefficient crosses the null (Fig. 2(b)). In sensitivity analyses, stratifying by (i) tenofovir disoproxil fumarate containing ART regimen and (ii) HIV viral load (≥ 60 copies/ml), we identified consistent evidence of an inflection point (at approximately 75 nmol/l) from the natural cubic spline models (online Supplementary Figs. 4(a) and 5(a)). Furthermore, a similar association between 25(OH)D and the vitamin D metabolic ratio was observed with an inflection point at 72·4 nmol/l (95 % CI: 67·1, 78·7) (online Supplementary 8(a)).

## Discussion

There is no global consensus for the clinical threshold value for defining vitamin D (25(OH)D) insufficiency or deficiency^(45,46)^. This study confirms the established non-linear association between total 25(OH)D and PTH and determines for the first time, using natural cubic spline modelling, a clear inflection point in the relationship between serum total 25(OH)D and PTH among children and adolescents living with HIV in southern Africa. This inflection point suggests that, in this population, a plasma total 25(OH)D of at least 75 nmol/l is required to see the 25(OH)D – PTH association levelling off. The inverse relationship between total 25(OH)D and PTH is strongest, at serum concentrations of 25(OH)D levels < 60 nmol/l. These thresholds appeared robust to ART regimen and HIV viral load.

A non-linear relationship between total 25(OH)D and PTH has been widely observed^(23,47)^, indicative of the role of low 25(OH)D contributing to increasing PTH concentrations in this population^(48)^. Unlike linear spline models^(22,49,50)^, which assume linearity leading to underfitting, the use of a natural cubic spline is more valid as (i) it allows for a non-linear association between 25(OH)D and PTH, which better fits the true relationship; (ii) can handle outliers for the association between 25(OH)D and PTH, by modelling the points with an additional constraint of linearity^(51)^; and (iii) can provide smoother flexible patterns showing different variations in the relationship between total 25(OH)D and PTH.

The present study suggests that vitamin D (25(OH)D) concentrations of at least 75 nmol/l may be required for PTH to be at its lowest among adolescents with HIV in southern Africa, in a setting with habitually low dietary Ca intakes. This aligns with the findings from other studies^(1,31)^ although many studies among HIV-negative populations have used lower 25(OH)D concentration to define vitamin D insufficiency (e.g. 50 nmol/l)^(31,52–55)^. Similar findings showing that levels of 25(OH)D > 75 nmol/l are required to lower PTH levels have also been reported in a healthy adult Kenyan population (*n* 253)^(56)^, a country with a comparable climate to Zambia and Zimbabwe, although levels were determined using a quadratic model.

Using the second polynomial function showed a less realistic symmetrical inverse relationship between 25(OH)D and PTH with the minimum point of the quadratic model as the inflection point. Despite the well-established non-linear relationship between total 25(OH)D and PTH^(56,57)^, some studies conducted in young people continue to use, arguably the less accurate, linear models to define 25(OH)D adequacy, and hence, results vary considerably^(22,56–60)^. As such, the Institute of Medicine’s recommendation to use 25(OH)D ≥ 50 nmol/l (20 ng/ml) is widely supported as an acceptable approach to prevent musculoskeletal disease among children and adolescents^(1,53)^. Studies of African populations, regardless of health status, have generally shown higher mean total 25(OH)D concentrations than in other regions globally^(20,61,62)^, as well as low dietary Ca intakes, as demonstrated in this analysis. Our findings that vitamin D (25(OH)D) concentrations of at least 75 nmol/l may be required for PTH to be at its lowest among adolescents with HIV in southern Africa raises the possibility that the Institute of Medicine’s recommended 50 nmol/l threshold to promote bone health might be too low in this setting.

The normal reference range for the liquid chromatography-tandem MS assay used to measure 1,25(OH)_2_D has been reported to be 108–246 pmol/l based on a population of Caucasian adolescents^(63)^. However, local 1,25(OH)_2_D reference values for African adolescents are limited and not available in the literature. In this study, 73 % of participants had a value that fell within this reference range, though the generalisability of this estimate is limited due to differences in sunlight exposure, dietary intake, genetic factors and skin pigmentation between the reference and study population.

This is the first study in East or Southern Africa to determine the association between 25(OH)D and PTH in adolescents living with HIV. Strengths include large sample size, the use of a robust non-linear model and the use of a common PTH assay with low levels of laboratory variation. However, the cross-sectional nature of the study prevents inference on causality between total 25(OH)D and PTH concentrations. Participants with secondary hyperparathyroidism were excluded as it would have been unethical to randomise them to the placebo-controlled trial. Heterogeneity in the data arises as a result of the inclusion of participants: (i) with different Ca intakes, (ii) at different pubertal stages, (iii) attending during different seasons and (iv) by combining boys and girls (as do all vitamin D clinical guideline recommendations), such that interpretation should be made bearing the population in mind. Dietary Ca intake was only semi-quantitatively assessed using a diet diversity questionnaire without direct validation against quantified portions, which may have underestimated intake. The relationship between total 25(OH)D and PTH is affected by multiple factors like ethnicity, pubertal status, renal function and dietary Ca intake, which was beyond the scope of this study to explore. Larger sample sizes, generating narrower CI, may identify an upper infection point > 75 nmol/l. The lack of an HIV-negative control group limited the generalisability of findings although it should be noted that in the impact of vertical HIV infection on child and adolescent skeletal development study in the Harare adolescent population^(64)^, Ca intakes were similarly low in HIV-negative control children.

In conclusion, this study reports an inverse relationship between total 25(OH)D and PTH in adolescents living with HIV and identifies inflection points at which the association changes; the association weakened when 25(OH)D exceeded 75 nmol/l. These results may be used to inform the epidemiology of vitamin D insufficiency in Southern Africa among individuals living with HIV. To what extent our findings are explained by the very low dietary Ca intake reported in this population, during a critical period of growth, merits further investigation. Ultimately, understanding the 25(OH)D-PTH relationship in greater detail is intended to help healthcare providers tailor appropriate supplementation strategies to improve bone health during a period of rapid growth and mineral accumulation in a nutritionally vulnerable population.

## Supporting information

Madanhire et al. supplementary material 1 Madanhire et al. supplementary material

Madanhire et al. supplementary material 2 Madanhire et al. supplementary material

Madanhire et al. supplementary material 3 Madanhire et al. supplementary material

Madanhire et al. supplementary material 4 Madanhire et al. supplementary material

Madanhire et al. supplementary material 5 Madanhire et al. supplementary material

Madanhire et al. supplementary material 6 Madanhire et al. supplementary material

Madanhire et al. supplementary material 7 Madanhire et al. supplementary material

Madanhire et al. supplementary material 8 Madanhire et al. supplementary material

Madanhire et al. supplementary material 9 Madanhire et al. supplementary material

## Supplementary material

For supplementary material/s referred to in this article, please visit https://doi.org/10.1017/S0007114525000509.
