# Supplementary figures and images for: The association between 25-hydroxyvitamin D and parathyroid hormone in adolescents living with HIV in southern Africa: a cross-sectional study
Source: Br J Nutr. 2025 Mar 13;133(7):885–91. doi: 10.1017/S0007114525000509 (PMC12198346; doi:10.1017/S0007114525000509)

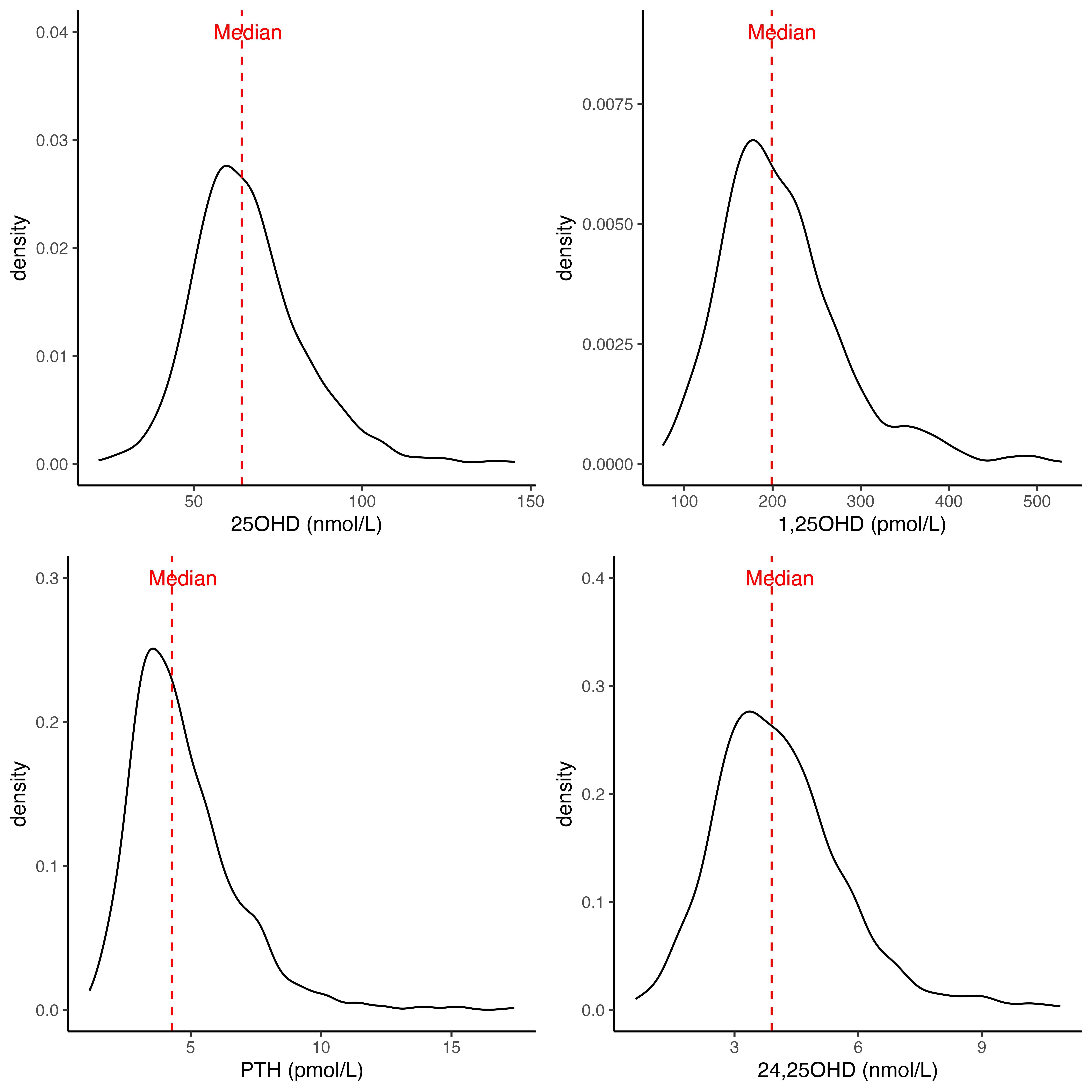

Supplement: Madanhire et al. supplementary material 1 — Madanhire et al. supplementary material [file S0007114525000509sup001.tiff]

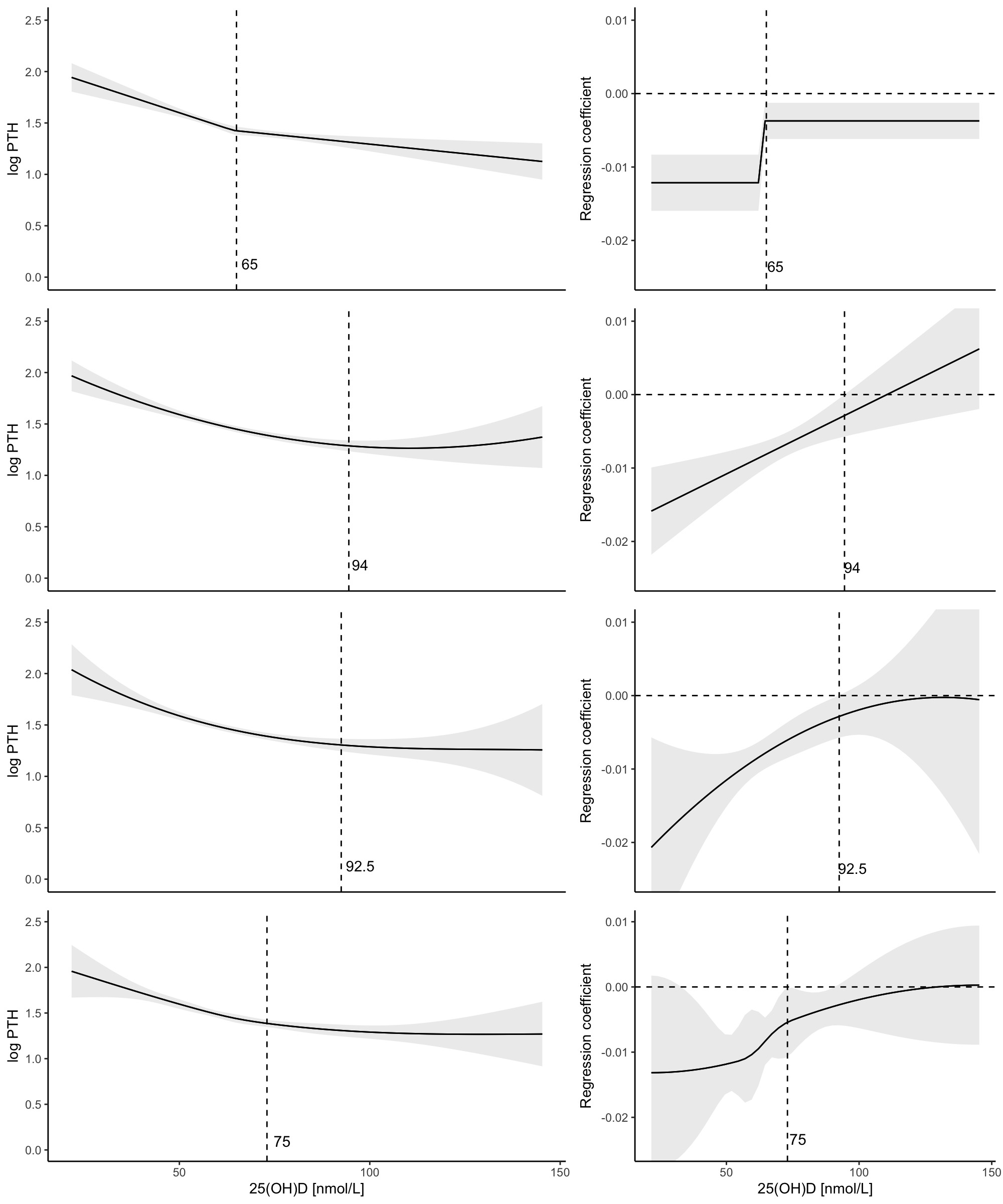

Supplement: Madanhire et al. supplementary material 2 — Madanhire et al. supplementary material [file S0007114525000509sup002.zip › Madanhire_BJN_Supp Figure 6a.jpeg]

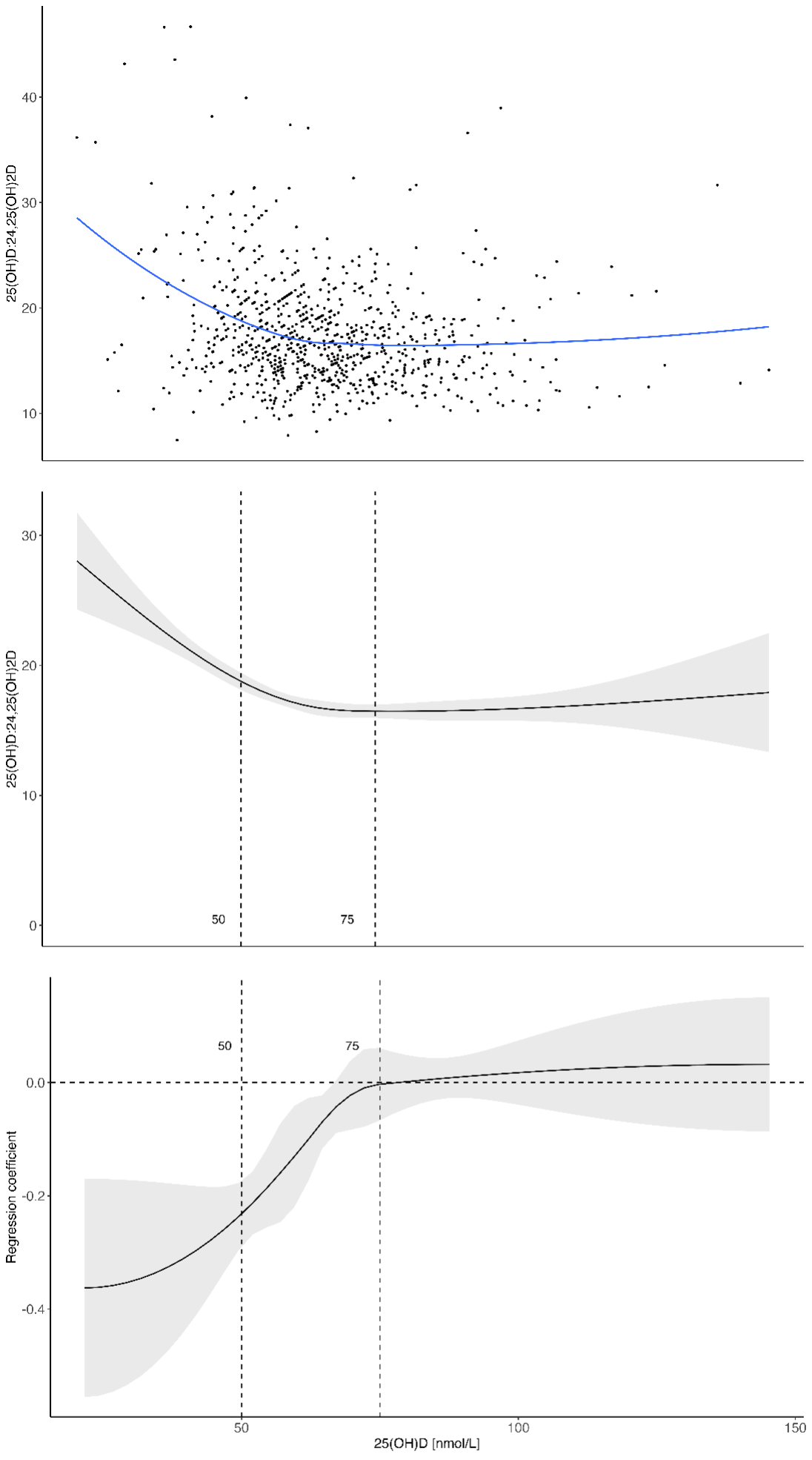

Supplement: Madanhire et al. supplementary material 3 — Madanhire et al. supplementary material [file S0007114525000509sup003.zip › Madanhire_BJN_Supplementary Figure 8a.png]

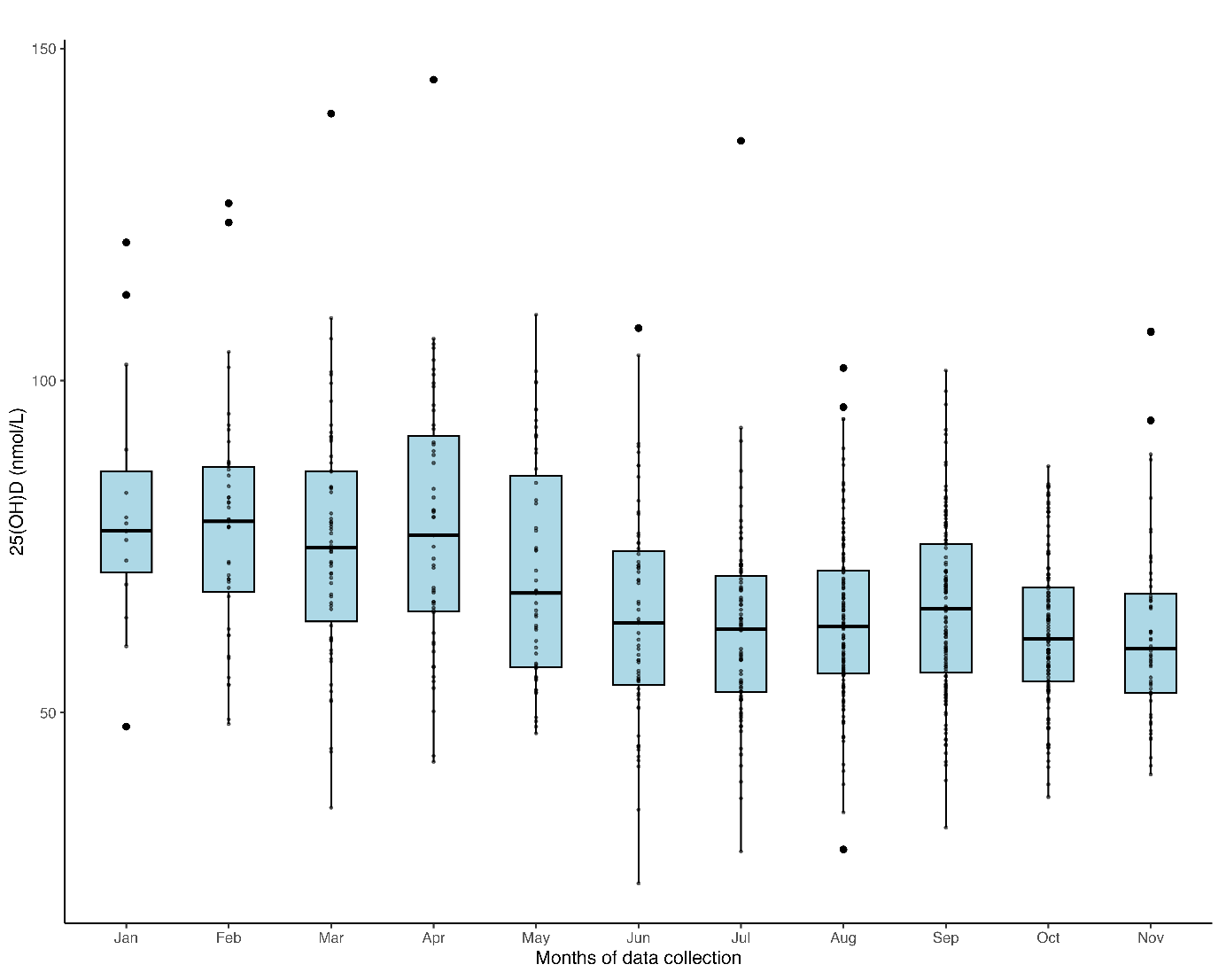

Supplement: Madanhire et al. supplementary material 4 — Madanhire et al. supplementary material [file S0007114525000509sup004.zip › Madanhire_BJN_Supplementary Figure 2a.png]

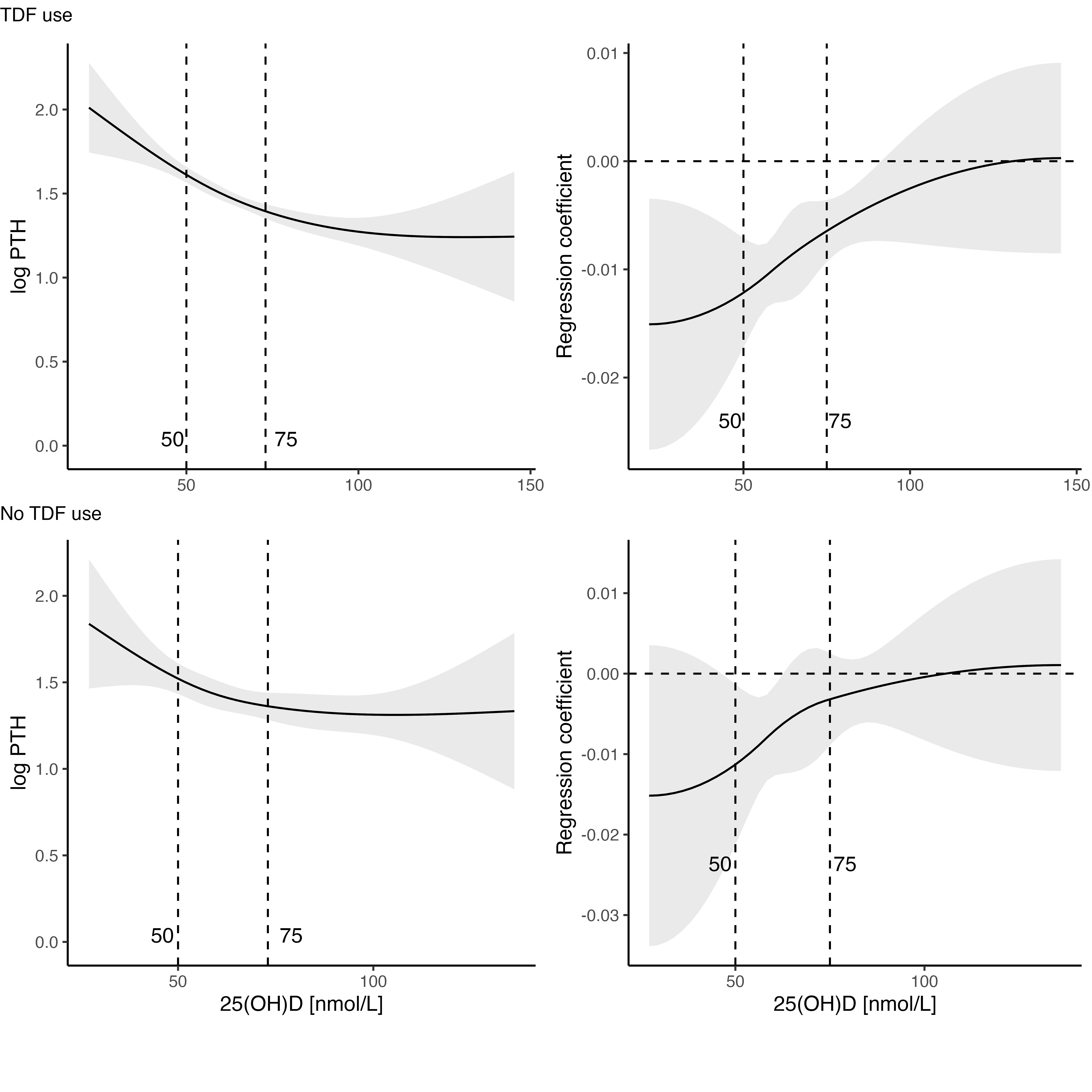

Supplement: Madanhire et al. supplementary material 5 — Madanhire et al. supplementary material [file S0007114525000509sup005.zip › Madanhire_BJN_Supplementary Figure 4a.jpg]

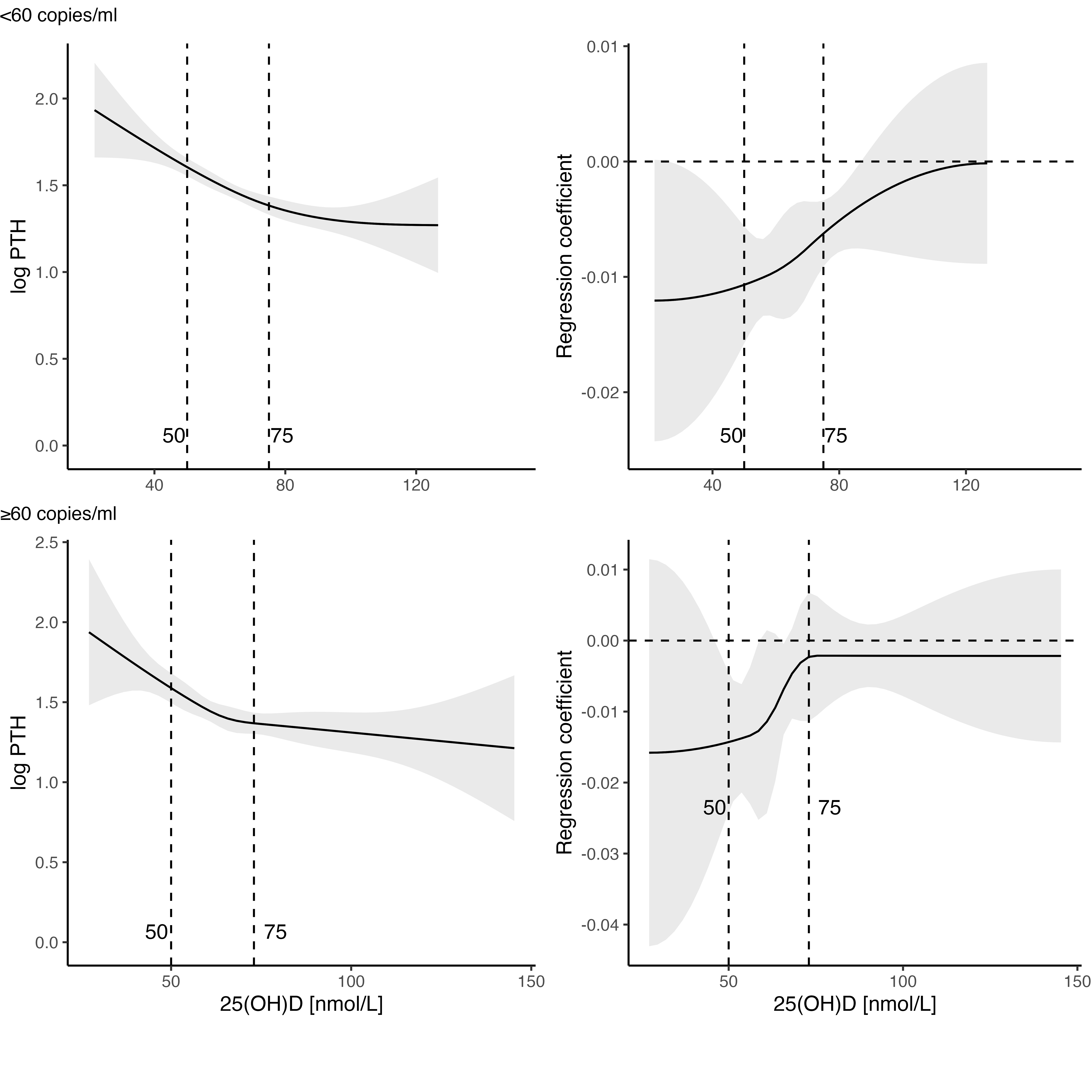

Supplement: Madanhire et al. supplementary material 6 — Madanhire et al. supplementary material [file S0007114525000509sup006.zip › Madanhire_BJN_Supplementary Figure 5a.jpg]

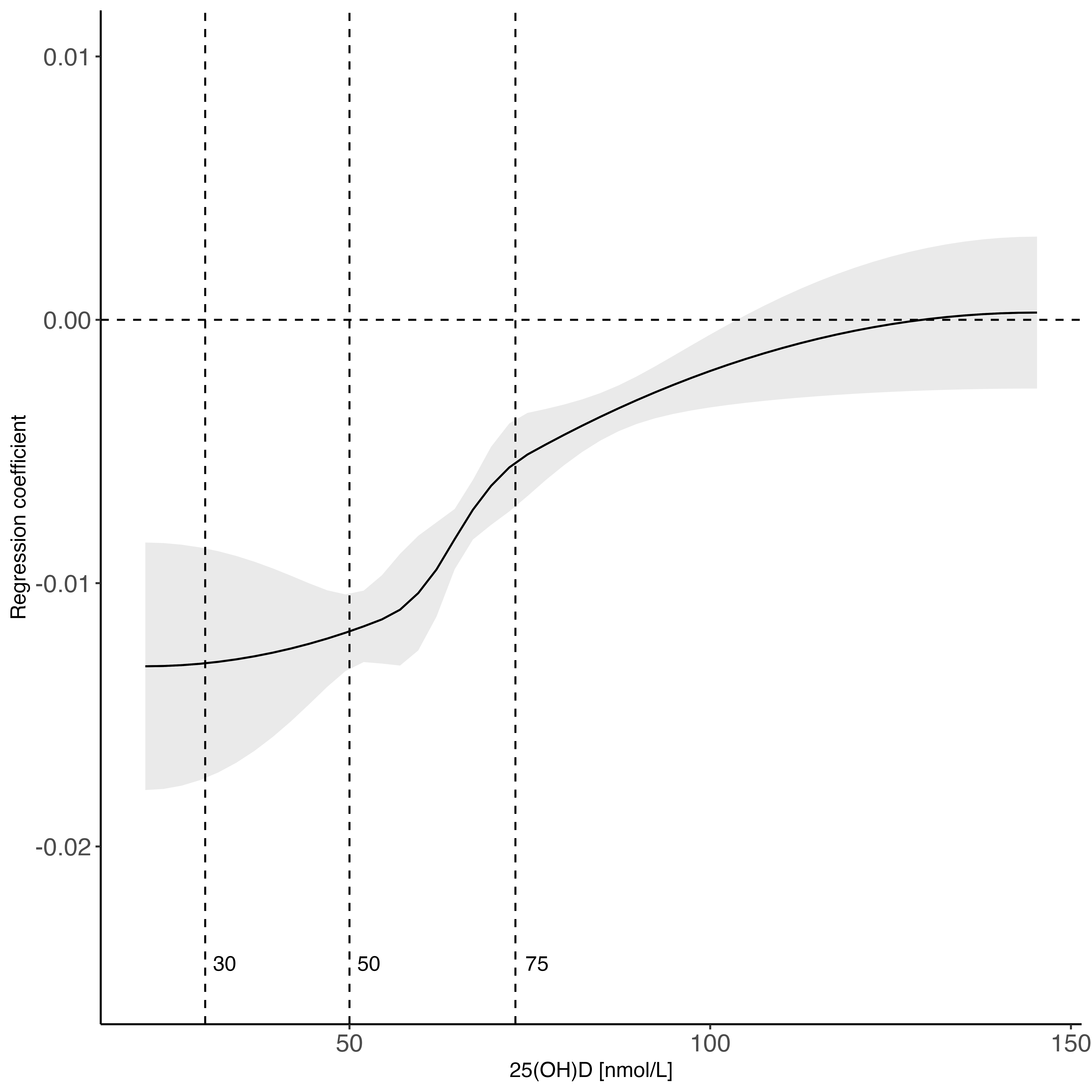

Supplement: Madanhire et al. supplementary material 7 — Madanhire et al. supplementary material [file S0007114525000509sup007.zip › Madanhire_BJN_Supplementary Figure 7a.jpg]

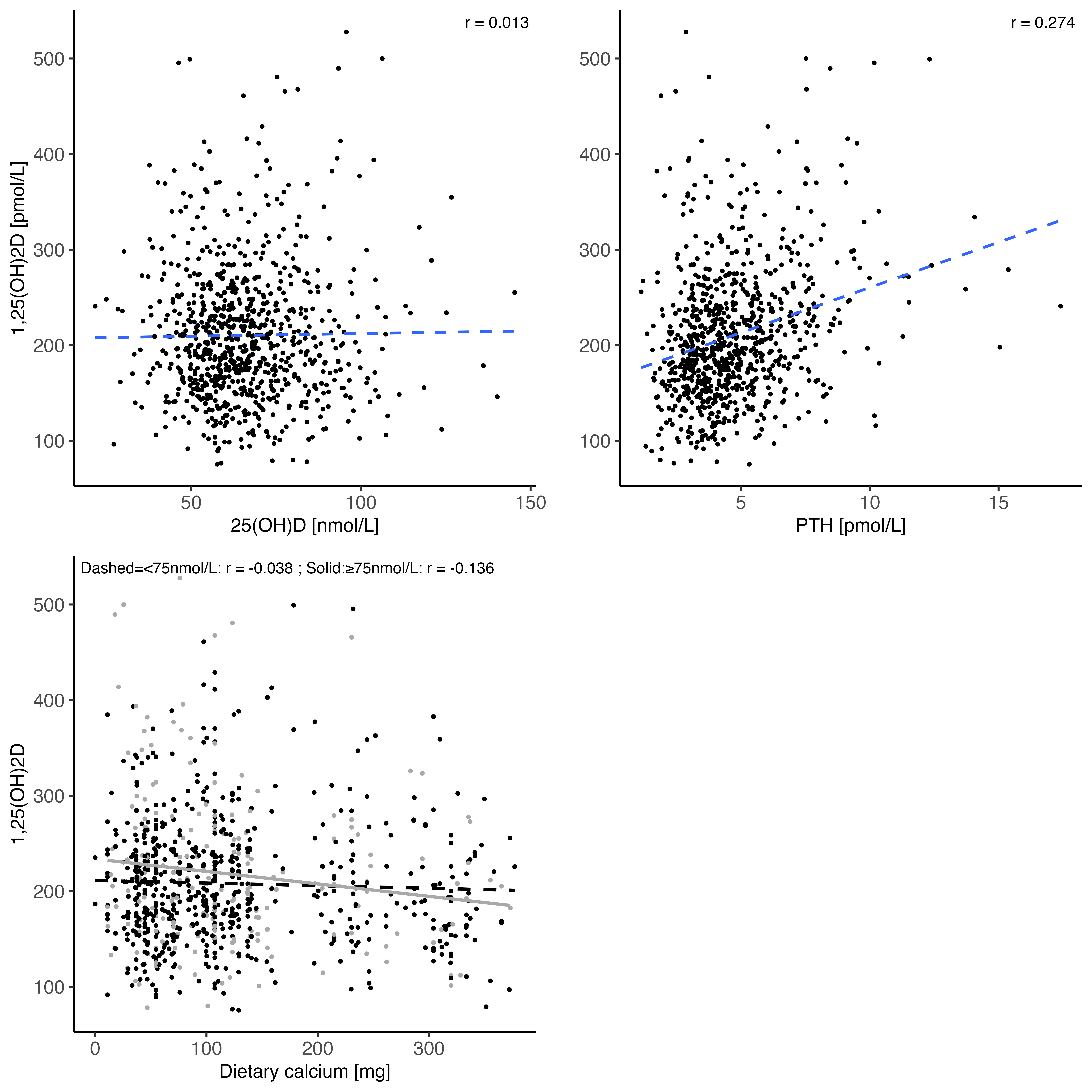

Supplement: Madanhire et al. supplementary material 8 — Madanhire et al. supplementary material [file S0007114525000509sup008.tiff]
