# Supplementary material for: The association between 25-hydroxyvitamin D and parathyroid hormone in adolescents living with HIV in southern Africa: a cross-sectional study
Source: Br J Nutr. 2025 Mar 13;133(7):885–91. doi: 10.1017/S0007114525000509 (PMC12198346; doi:10.1017/S0007114525000509)
Supplement: Madanhire et al. supplementary material 9 — Madanhire et al. supplementary material [file S0007114525000509sup009.docx]

**The association between 25-hydroxyvitamin D and parathyroid hormone in adolescents living with HIV in southern Africa: a cross sectional study**

**Supplementary table 1: Lower and upper limits for weekly dietary calcium intake (mg/week) for different food sources and frequencies**

|  | **Calcium Intake-Lower Limit (mg/week)** | **Calcium Intake-Upper Limit (mg/week)** |
| --- | --- | --- |
| **Dairy** | | |
| Never | 0 | 0 |
| Less than 4 times in past month | 10 | 249 |
| 1-2 times a week | 250 | 749 |
| 3-5 times a week | 750 | 1749 |
| Almost every day | 1750 | 2000 |
| **Legumes** | | |
| Never | 0 | 0 |
| Less than 4 times in past month | 10 | 39 |
| 1-2 times a week | 40 | 119 |
| 3-5 times a week | 120 | 279 |
| Almost every day | 280 | 320 |
| **Fish** | | |
| Never | 0 | 0 |
| Less than 4 times in past month | 10 | 49 |
| 1-2 times a week | 50 | 149 |
| 3-5 times a week | 150 | 349 |
| Almost every day | 350 | 400 |
| **Eggs** | | |
| Never | 0 | 0 |
| Less than 4 times in past month | 10 | 37 |
| 1-2 times a week | 38 | 110 |
| 3-5 times a week | 111 | 258 |
| Almost every day | 259 | 304 |
| **References:**  Calcium calculations:  <https://www.iofbonehealth.org/calcium-calculator>  <https://www.nof.org/patients/treatment/calciumvitamin-d/a-guide-to-calcium-rich-foods/> | | |

*Calcium estimates are based on the International Osteoporotic Foundation (IOF) calculator after considering dairy, legumes, fish and eggs (micronutrient rich foods as per Food and Agriculture Organization of the United Nations questionnaire). Lower and upper limits for calcium intake per food item (mg/week) are calculated based on serving size, calcium quantity/serving and weekly intake frequency.*

**Supplementary table 2: Median age, dietary calcium intake, serum PTH and 1,25(OH)_2_D concentrations by Tanner stage and sex**

| **n (%)** | **Tanner**  **stage** | **Age (years),** *median (IQR)* | **Dietary calcium (mg),** *median (IQR)* | **PTH (pmol/L),** *median (IQR)* | **1,25(OH)_2_D (pmol/L),** *median (IQR)* |
| --- | --- | --- | --- | --- | --- |
| **Females** | | | | | |
| 43 (9.6) | I | 11.6 [11.3, 12.1] | 90.3 [47.4, 139.1] | 4.1 [3.1, 5.6] | 221.4 [176.4, 269.2] |
| 45 (10.1) | II | 12.2 [11.4, 13.1] | 100.3 [60.4, 161.8] | 4.3 [3.6, 6.1] | 213.2 [186.2, 255.6] |
| 76 (17.0) | III | 12.9 [12.0, 14.2] | 107.5 [54.6, 205.7] | 4.6 [3.5, 6.8] | 223.7 [179.9, 264.3] |
| 113 (25.3) | IV | 14.9 [13.7, 16.3] | 92.8 [47.4, 140.4] | 4.4 [3.2, 5.7] | 209.9 [172.8, 258.9] |
| 170 (38.0) | V | 17.6 [16.2, 18.6] | 107.5 [62.6, 220.4] | 4.3 [3.3, 5.4] | 175.8 [148.3, 210.5] |
| **Males** | | | | | |
| 34 (8.7) | I | 11.7 [11.3, 12.6] | 86.0 [54.6, 126.2] | 3.9 [2.9, 5.4] | 195.9 [151.4, 227.1] |
| 84 (21.4) | II | 12.6 [12.1, 13.6] | 88.7 [48.9, 152.3] | 3.8 [3.1, 4.7] | 190.8 [162.1, 236.1] |
| 90 (22.9) | III | 14.7 [13.7, 16.0] | 105.7 [65.8, 131.8] | 4.3 [3.3, 5.7] | 226.8 [180.6, 277.7] |
| 94 (23.9) | IV | 16.4 [15.1, 17.6] | 84.8 [52.6, 140.4] | 4.4 [3.4, 6.3] | 211.0 [174.2, 255.8] |
| 91 (23.2) | V | 18.4 [17.6, 19.1] | 103.3 [53.8, 139.7] | 4.5 [3.4, 6.3] | 182.8 [144.3, 235.1] |
